# The Xavier Ochsner College of Medicine – “The Time Is Always Right To Do What Is Right”*

**DOI:** 10.31486/toj.24.5049

**Published:** 2024

**Authors:** Yvens Laborde

**Affiliations:** Global Health and Education, Ochsner Clinic Foundation, New Orleans, LA and The University of Queensland Medical School, Ochsner Clinical School, New Orleans, LA

## THE PROBLEM

We have a shortage of Black doctors in the United States. According to the 2022 U.S. Physician Workforce Data Report, Blacks make up 5.2% of all practicing physicians in the United States,^1^ while the 2020 Census estimated that Blacks make up 13.7% of the total population.^2^ In Louisiana, Blacks make up 32.6% of the population,^2^ but only 8.3% of the practicing physicians in the state are Black.^3^ Data suggest that this lack of representation is a major contributor to the historically poor health outcomes for Black patients.^4^

Exacerbating the low percentage of Black physicians in Louisiana is the projected shortage of physicians overall in the state. By 2030, Louisiana is projected to have a shortage of 4,820 doctors.^5^ Currently, 60 of Louisiana's 64 parishes are health professional shortage areas (HPSAs), meaning that those parishes have 3,500 or more patients for every provider.^5^ Almost 2 million Louisiana residents live in an HPSA,^5^ so it is no accident that Louisiana ranked last—#50—in the United Health Foundation 2023 annual report of America's Health Rankings.^6^

Health disparities are not just a problem in Louisiana. Caraballo et al examined US national data from the Centers for Disease Control and Prevention for the 22-year period 1999 through 2020 for non-Hispanic White and non-Hispanic Black populations across all age groups. Their analysis showed that the Black population in the United States experienced more than 1.63 million excess deaths and more than 80 million excess years of life lost compared with the White population.^7^

## THE HISTORIC CONTEXT

In the late 1800s and early 1900s, at least 14 medical schools for Black students were established in the United States, principally in the South. These medical schools were seen as a solution to the racialized health care system that persisted in the United States after the end of the Civil War, and the hope was that the doctors these schools produced would practice in their communities and provide care for those that society chose to ignore and discriminate against.

While racist national, state, and local policies contributed to the decline of many of these schools throughout the Jim Crow era, the most influential factor in the demise of Black medical schools was Abraham Flexner. In 1904, the Carnegie Foundation established the Council on Medical Education and in 1908 tasked Flexner to assess medical schools in the United States and Canada and to provide recommendations for streamlining and raising the standards of medical education. At that time, medical education was not regulated, and few state licensing laws existed. The objective of the Council on Medical Education was to restructure and standardize medical education.

In 1910, after traveling to every medical school in North America, Flexner produced *Medical Education in the United States and Canada: A Report to the Carnegie Foundation for the Advancement of Teaching*, a document now known as the Flexner Report,^8^ that became an essential part of US medical history. The Flexner Report created the context for how we understand medical education and how medical education in the United States is structured.^9^

One of Flexner's major concerns was the financial support and viability of medical schools and guarding against “cheaply made doctors.”^9^ He argued that the country needed “fewer and better doctors,” concluding that southern states only needed 1,300 physicians and current enrollment far exceeded demand.^9^ He reasoned that only 6 medical schools were sufficient to produce the physicians needed to take care of the southern population.^9^ However, Flexner's figures only applied to the number of physicians needed to take care of the South's White population, and the 6 schools he determined were worth keeping did not include the Black medical schools.^9^

Only 7 Black medical schools were still in operation at the time of Flexner's assessment, and he wrote, “Of the seven medical schools for negroes in the United States, five are at this moment in no position to make any contribution of value…; Flint [Medical College] at New Orleans, Leonard [Medical School] at Raleigh, the Knoxville [Medical College], Memphis [Medical Department of the University of West Tennessee], and Louisville [National Medical College] schools are ineffectual.”^8^ Flexner deemed that “Meharry [Medical College] at Nashville and Howard [University] at Washington are worth developing…” and explained this decision by writing, “The negro must be educated not only for his sake, but for ours,” having pointed out that “Ten million of them live in close contact with sixty million whites. Not only does the negro himself suffer from hookworm and tuberculosis; he communicates them to his white neighbors….”^8^

Consequently, in 2024, there are only 4 historically Black college and university (HBCU) medical schools in the United States: Howard University College of Medicine (founded in 1868), Meharry Medical College (founded in 1876), Charles R. Drew University of Medicine and Science (founded in 1966), and Morehouse School of Medicine (founded in 1975).^10^ A fifth HBCU medical school is scheduled to open in fall 2026: the Maryland College of Osteopathic Medicine at Morgan State University in Baltimore, Maryland.^11^

Graduates of the 4 HBCU medical schools make up approximately 50% of the nation's Black physicians, while representing just 2% of the nation's 193 medical schools.^10^ The HBCU medical school model is a successful model. A 2023 study showed that Black medical students attending HBCU medical schools reported a greater sense of belonging and higher confidence in their scholastic abilities than Black students in predominantly White medical schools.^12^

The schools that still exist are punching above their weight and if others had survived, the US physician workforce would more accurately reflect patient diversity. A 2020 economic evaluation showed that if the 5 additional schools had remained open after the release of the Flexner Report, those schools might have produced an additional 35,315 graduates by 2019, increasing the number of Black physicians by 29%.^13^ While much has changed to improve health equity in the United States since the end of the Jim Crow era, many of the health inequities that the early Black medical schools were created to address persist today.

## THE SOLUTION

Ochsner Health in New Orleans, Louisiana, is committed to addressing health inequities and improving physician diversity. Xavier University of Louisiana, in partnership with Ochsner Health, is establishing a new HBCU medical school, aiming to replicate the success of existing HBCUs in producing competent, culturally aware physicians who can serve the health needs of a diverse population. The Xavier Ochsner College of Medicine team is working with the Liaison Committee on Medical Education to obtain preliminary accreditation status.

In a 2023 study, Snyder et al reported higher life expectancy and better health outcomes among the Black population in counties served by Black primary care physicians.^4^ Torres^14^ and Alsan et al^15^ showed improved rates of preventive care compliance and outcomes in patients with racially concordant physicians. Greenwood et al showed that Black babies and mothers cared for by Black doctors have better health and mortality outcomes.^16^

The lack of representation is an urgent matter of life, death, and restorative justice. Creating opportunities to train and graduate more minority physicians will be good for the health and prosperity of the entire nation.

The Xavier Ochsner College of Medicine is an example of the type of initiative that holds true promise for providing real-world solutions to the shortage of underrepresented physicians and to the shortage of physicians overall in Louisiana. The Xavier-Ochsner partnership provides a blueprint for how other predominantly White institutions can partner with HBCUs to address this regional and national problem.

## CONCLUSION

Malcolm X understood that “Education is our passport to the future, for tomorrow belongs only to the people who prepare for it today.”^17^ The Xavier Ochsner College of Medicine is an innovative partnership that will create a medical school designed to produce the high-quality physicians that our diverse communities so desperately need now and in the future.
